# Rb and p53 Liver Functions Are Essential for Xenobiotic Metabolism and Tumor Suppression

**DOI:** 10.1371/journal.pone.0150064

**Published:** 2016-03-11

**Authors:** Sathidpak Nantasanti, Mathilda J. M. Toussaint, Sameh A. Youssef, Peter C. J. Tooten, Alain de Bruin

**Affiliations:** 1 Department of Pathobiology, Faculty of Veterinary Medicine, Utrecht University, 3584CL, Utrecht, the Netherlands; 2 Department of Pediatrics, Division of Molecular Genetics, University Medical Center Groningen, University of Groningen, 9713 AV, Groningen, the Netherlands; University of Navarra School of Medicine and Center for Applied Medical Research (CIMA), SPAIN

## Abstract

The tumor suppressors Retinoblastoma (Rb) and p53 are frequently inactivated in liver diseases, such as hepatocellular carcinomas (HCC) or infections with Hepatitis B or C viruses. Here, we discovered a novel role for Rb and p53 in xenobiotic metabolism, which represent a key function of the liver for metabolizing therapeutic drugs or toxins. We demonstrate that Rb and p53 cooperate to metabolize the xenobiotic 3,5-diethoxycarbonyl-1,4-dihydrocollidine (DDC). DDC is metabolized mainly by cytochrome P450 (Cyp)3a enzymes resulting in inhibition of heme synthesis and accumulation of protoporphyrin, an intermediate of heme pathway. Protoporphyrin accumulation causes bile injury and ductular reaction. We show that loss of Rb and p53 resulted in reduced Cyp3a expression decreased accumulation of protoporphyrin and consequently less ductular reaction in livers of mice fed with DDC for 3 weeks. These findings provide strong evidence that synergistic functions of Rb and p53 are essential for metabolism of DDC. Because Rb and p53 functions are frequently disabled in liver diseases, our results suggest that liver patients might have altered ability to remove toxins or properly metabolize therapeutic drugs. Strikingly the reduced biliary injury towards the oxidative stress inducer DCC was accompanied by enhanced hepatocellular injury and formation of HCCs in Rb and p53 deficient livers. The increase in hepatocellular injury might be related to reduce protoporphyrin accumulation, because protoporphrin is well known for its anti-oxidative activity. Furthermore our results indicate that Rb and p53 not only function as tumor suppressors in response to carcinogenic injury, but also in response to non-carcinogenic injury such as DDC.

## Introduction

Xenobiotics are compounds originating from outside the body, such as drugs and toxins. Once entered into the mammalian body, xenobiotics get removed by xenobiotic metabolism, which occurs predominately in the liver and to some extend in the intestine. Cytochrome P450 enzymes (Cyps) are the key components for oxidation and associated detoxification of xenobiotics [1]. The goal is to detoxify these foreign chemical species and convert them into molecules that are soluble and readily excreted. The large substrate-binding cavities of Cyps allow them to accommodate a wide range of substrates, most of which are hydrophobic [2]. In some cases the Cyp activity can be altered by interaction with the xenobiotics. Since modification in Cyps activity will influence the metabolism and detoxification of xenobiotics, identifying factors that change Cyps expression will be important for mammals exposed to toxins and for patients receiving drugs that cause severe side effects or have a small therapeutic window [3].

Recent studies provide some evidence that expression of Cyps can be regulate by the tumor suppressor genes Retinoblastoma (Rb) and p53 [3,4], however the consequences for metabolism of xenobiotics in livers are unknown. Both Rb and p53 are frequently inactivated in the liver, because of infection with Hepatitis B or C viruses. Both type of viruses produces viral proteins that can bind to these tumor suppressors and thereby inhibit its functions [5–8]. Furthermore genetic analysis of HCC revealed that a subset of liver tumor carry Rb and p53 mutations [5]. Rb and p53 play crucial roles as cell cycle regulators [9]. Apart for their role in cell-cycle control, Rb and p53 have been implicated in many cellular processes including DNA-damage responses, DNA repair, DNA replication, cellular differentiation and apoptosis [10].

The Rb protein and its family members, p107 and p130, mediate cellular response through the control of E2F transcription factor activity and the expression of their target genes during cell cycle progression. Under resting condition or G0 phase, hypophosphorylated pRB binds and inhibits E2F transactivation domain. In proliferative stage, pRB is inactivated by Cyclin-CDK (cyclin-dependent kinase) complexes and becomes hyperphophorylated. This inactive pRB then releases E2F to allow the induction of genes involve in the transition from G1 phase to S phase of cell cycle. Therefore, the loss or mutation of Rb leads to deregulation of E2F and inappropriate proliferation which can contribute to cancer development [10,11]. One pathway likely to mediate cell cycle control in the absence of Rb is the p53 pathway [4]. In response to cellular stress, the transcription factor p53 is phosphorylated and binds to specific target genes involved in DNA repair, cell cycle progression, and apoptosis [9,12]. Multiple mechanism have been discovered that resulted in inactivation of the p53 pathway in HCC [13,14].

Although combined loss of Rb and p53 is a frequent event in human cancer, and many different cancer patients are treated with xenobiotic anti-cancer drugs, the impact of Rb and p53 loss on xenobiotic metabolism is unknown. To determine the role of Rb and p53 in xenobiotic metabolism, we exposed mice with liver-specific ablation of Rb and p53 to the xenobiotic 3,5-diethoxycarbonyl-1,4-dihydrocollidine (DDC). Exposure of DDC to mice is an established xenobiotic-induced mouse model of porphyrias, a disease where excess amounts of protoporphyrin accumulate in the body. Protoporphyrin is required for synthesis of heme, which carries the oxygen in the blood. DDC interacts directly with Cyps, in particular Cyp3a [15], leading to the generation of an inhibitor of ferrochelatase, a key enzyme required for heme biosynthesis pathway. Inhibition of ferrochelatase causes accumulation of its substrate protoporphyrin especially in bile ducts triggering cholangitis and ductular reactions [15].

Here, we used the DDC mouse model, and demonstrate that combined deletion of Rb and p53 in the liver reduces Cyp3a expression and DDC metabolism, which led to less accumulation of DDC-metabolite protoporphyrin and less bile duct injury. Moreover, we discovered that hepatocellular injury was instead increased and HCC developed in Rb and p53 deficient livers in response to DDC. These findings show that Rb and p53 function are required for efficient metabolism of the xenobiotic DDC and tumor suppression in the liver.

## Materials and Methods

### Animals

The albumin (alb) enhancer promoter and cre recombinase/*lox*P system were used to generate conditional liver specific Rb and/or p53 knockout mice. The alb gene promoter provides specific deletion of the gene of interest in the liver, as alb is expressed mainly in hepatocytes and bile duct cells [16]. Cre recombinase facilitates the excision of target gene which flanked by two *lox*P sites [17]. In this study, mice expressing Cre recombinase under the control of albumin [18] were crossed with mice harboring Rb (Rb^f/f^) and/or p53 (p53^f/f^) flanked by two *loxP* sites [19]. All mice were bred at least in 7^th^ generations FVB. Genotyping was performed by polymerase chain reaction on DNA isolated from ear cuts.

### Treatment

8–9 weeks old, male and female mice were fed ad libitum with diet contained 0.1% DDC (Bio-serv, New Jersey, USA) for 3 weeks, and then the mice were harvested (5 mice per group) or changed to normal diet for life span analysis (5–11 mice per group). Control littermate mice were fed a standard mouse diet. No differences were observed between male and female in the responses to DDC. All mice were housed under standard laboratory conditions. During DDC feeding and survival study, health status and body weight of the mice were monitored 2–3 times a week. All mice survived 3 weeks DDC feeding. The mice were euthanized using CO_2_ inhalation. For the life span studies mice were euthanized when they fulfilled one of the following human end point criteria: (1) more than 10% change in body weight within one week (2) abnormal behaviors (not response to stimulus, continuous tremble, self-mutilation) (3) piloerection longer than 24 hours (4) abnormal enlargement of abdomen (5) labored respiration (6) loss of ability to ambulate. Experiments were performed in accordance with institutional and national guidelines approved by Utrecht University Animal Ethics Committee.

### Protoporphyrin Measurement

Unstained liver sections were photographed under polarization light to quantify the accumulation of fluorescent protoporphyrin. Adobe photoshop was used to switch the fluorescent protoporphyrin to black color, ImageJ software was then applied to quantify protoporphyrin area. At least 20 randomized liver regions per mouse were analyzed.

### Serum Biochemistry

Blood samples were collected from inferior vena cava before removing the liver. Blood samples were centrifuged to collect serum. Serum alanine aminotransferase (ALT) and serum aspartate aminotransferase (AST) were measured using the UniCel DxC Synchrome (Beckman Coulter, Woerden, NL).

### Statistical Analysis

All experimental data are shown as mean ± standard error of mean (SEM). The statistics analyses for bar graphs were performed with SPSS. P-values were set at P < 0.05 and calculated using Kruskal Wallis and Mann-Whitney U test. Survival curve was drawn and statistical difference was calculated using SPSS log rank test.

Additional information on methods is provided in S1 File: Materials and Methods.

## Results

### Combined loss of Rb and p53 suppresses protoporphyrin accumulation and ductular reaction in livers of mice fed with DDC

Previous studies have demonstrated that expression of genes involved in xenobiotic metabolism are reduced in Rb and Rb/p53 deficient cells [3,4]. To determine *in vivo* whether Rb and p53 functions are required for metabolism of xenobiotics, we fed mice a diet containing the xenobiotic DDC. Since DDC is metabolized in the liver, we generated mice that are deficient for *Rb* and *p53* in the liver. To this end, conditional Rb^f/f^ and p53^f/f^ mice were crossed with transgenic mice expressing the cre-recombinase under the liver-specific albumin enhancer promoter to obtain the deletion of Rb and p53 function in mouse livers. The endogenous albumin gene is expressed during embryonic development in liver progenitor cells which then give rise to hepatocytes and bile duct cells [20,21], leading to efficient deletion of *Rb* and *p53* in those three parenchymal liver cell types of adult mice. Genotyping PCR of liver sections confirmed the deletion of Rb and p53 (S1 Fig).

**Fig 1:**
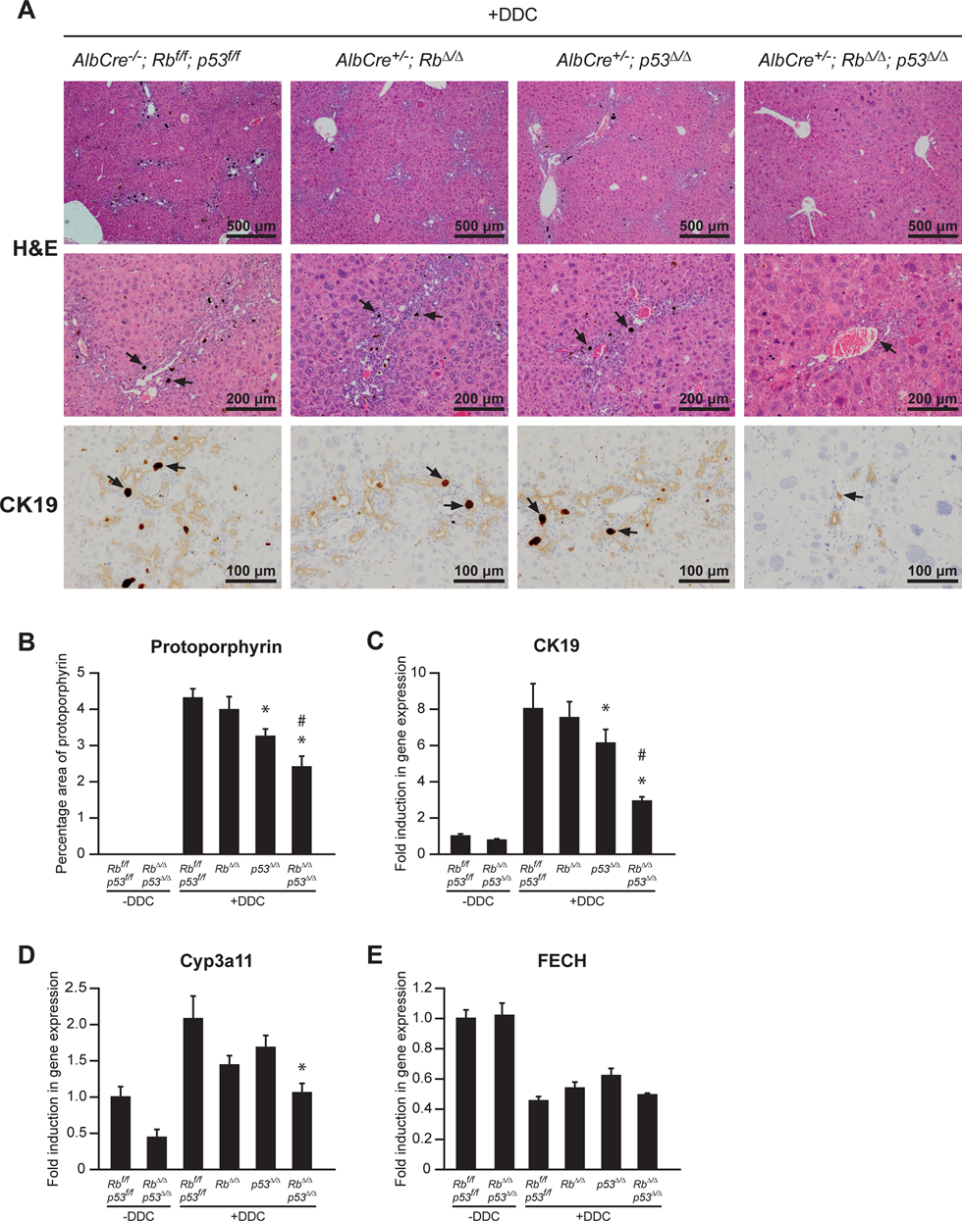
Combined loss of Rb and p53 suppresses protoporphyrin accumulation, ductular reaction and Cyp3a expression in livers of DDC-fed mice. (**A**) Representative pictures of H&E and CK19 staining on liver sections from mice of indicated genotypes after 3 weeks of DDC feeding. Arrows indicate protoporphyrin accumulation. (**B**) Semi-quantitative analysis of percentage area of protoporphyrin accumulation from mice fed with normal chow or 3-weeks DDC diet (n = 5 per group). (**C**) (**D**) (**E**) Expression of *CK19*, *Cyp3a11* and *FECH* was measured by qPCR in livers of indicated genotypes. -DDC, *Rb*^*f/ f*^*; p53*^*f/f*^, were normalized to 1. Data presented as average ± SEM. * P < 0.05 compared to +DDC, *Rb*^*f/ f*^*; p53*^*f/f*^, # P < 0.05 compared to +DDC, *Rb*^Δ/Δ^.

Adult wild-type mice (*Wt*: *Alb*^*-/-*^*; Rb*^*f/f*^*;p53*^*f/f*^), mice with liver-specific deletion of *Rb*^Δ/Δ^ (Alb^+/-^; *Rb*^Δ/Δ^), mice with liver-specific deletion of *p53*^Δ/Δ^ (Alb^+/-^; *p53*^Δ/Δ^), and mice with liver-specific combined deletion of *Rb*^Δ/Δ^;*p53*^Δ/Δ^ (Alb^+/-^; *Rb*^Δ/Δ^; *p53*^Δ/Δ^) were fed a diet containing 0.1% DDC for 3 weeks. All the mice fed with DDC developed yellowish mucous membrane and the color of livers was clearly darker compared to untreated groups (S2A Fig). Histological analysis of hematoxylin and eosin stained liver sections of DDC-treated mice revealed that *Wt*, *Rb*^Δ/Δ^ and *p53*^Δ/Δ^ livers displayed strong accumulation of intraductal dark-brown protoporphyrin pigment plugs accompanied with ductular reactions near portal regions (Fig 1A). In contrast, protoporphyrin accumulation and ductular reaction were markedly reduced in *Rb*^Δ/Δ^;*p53*^Δ/Δ^ livers. Quantitative analysis of protoporphyrin accumulation by examining unstained liver slides under polarizing light confirmed the reduced accumulation of protoporphyrin in *Rb*^Δ/Δ^;*p53*^Δ/Δ^ livers (Fig 1B). The protoporphyrin accumulation within the bile ducts provokes a ductular reaction leading to the formation of smaller bile ducts through proliferation of liver progenitor cells (oval cells). In addition, this ductular reaction is accompanied with a ccumulation of hepatic stellate cells, biliary fibroblasts, and inflammatory cells such as macrophages and neutrophils. We have performed immunohistochemical analysis with markers for oval/bile duct cells (CK19), hepatic stellate cells (GFAP), and macrophages (F4/80), and verified that the number of oval cells, macrophages and hepatic stellate cells were markedly reduced in *Rb*^Δ/Δ^;*p53*^Δ/Δ^ livers compared to other genotypes (Fig 1A; S3A and S3B Fig). These findings were also supported by quantitative PCR analysis for *CK19* (Fig 1C). The baseline *CK19* mRNA levels were not different between *Wt* and *Rb*^Δ/Δ^;*p53*^Δ/Δ^ untreated mice. After DDC treatment, *CK19* mRNA levels increased 6-8-fold in *Wt*, *Rb*^Δ/Δ^ and *p53*^Δ/Δ^ livers, whereas *Rb*^Δ/Δ^;*p53*^Δ/Δ^ livers showed only a 2-fold increase in *CK19* expression. Together these findings demonstrate that loss of Rb and p53 in the liver suppresses protoporphyrin accumulation and ductular reaction in mice fed with DDC.

### Cyp3a expression is reduced in *Rb*^Δ/Δ^;*p53*^Δ/Δ^ livers after DDC feeding

To explore why loss of Rb and p53 mitigates DDC-induced protoporphyrin accumulation in the liver, we first evaluated whether Cyp3a expression, the enzyme that mainly metabolizes DDC, was altered. We performed qPCR analysis, and discovered that *Cyp3a11* mRNA levels were induced in *Wt* livers upon DDC exposure (Fig 1D). Importantly, *Rb*^Δ/Δ^;*p53*^Δ/Δ^ livers show marked reduction in Cyp3a expression compared to *Wt* livers. Inactivation of Rb or p53 alone reduced Cyp3a expression as well, but to a lesser extent than the combinatorial knockout. These findings suggest that loss of Rb and p53 reduces Cyp3a expression and thereby inhibiting DDC metabolism and protoporphyrin accumulation. Next, we investigated whether the expression levels of ferrochelatase, the enzyme that converts protoporphyrin into heme [22], were changed in *Rb*^Δ/Δ^;*p53*^Δ/Δ^ livers. However, mRNA levels of *ferrochelatase* (*FECH*) did not differ between genotypes (Fig 1E), indicating that the observed phenotypes were most likely not related to alterations in ferrochelatase expression.

### Loss of Rb and p53 results in increased hepatocellular injury after DDC exposure

**Fig 2:**
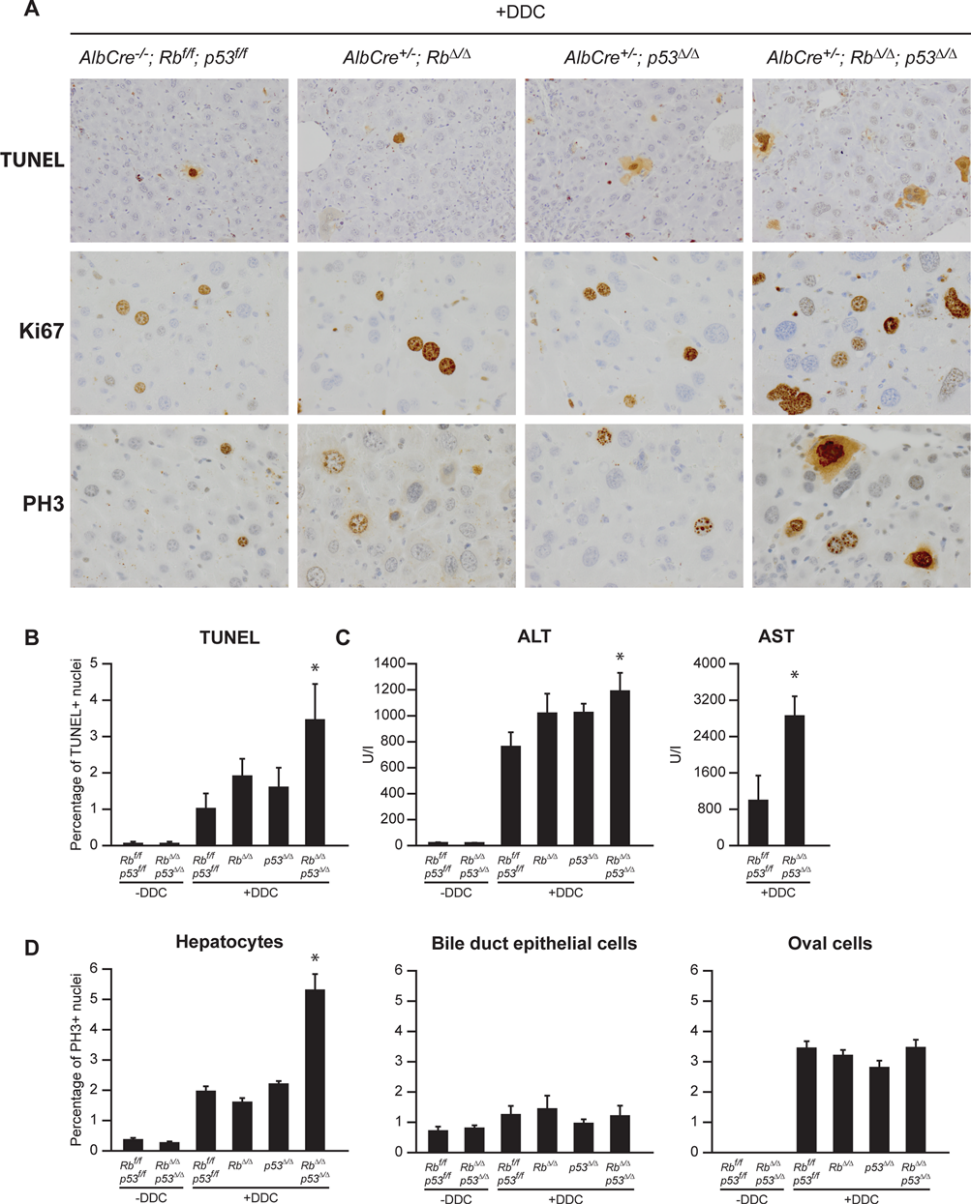
Loss of Rb and p53 results in increased hepatocellular injury, hepatocyte proliferation and polyploidization after DDC. (**A**) ALT and AST serum levels (**B**) Representative pictures of TUNEL, Ki67 and PH3 staining on liver sections (**C**) Percentage of TUNEL positive hepatocyte nuclei (**D**) Percentage of PH3 positive hepatocyte nuclei (**E**) Mitotic index of hepatocytes (**F**) Multinucleated hepatocytes (G) Anisokaryosis score of hepatocytes from mice of indicated genotypes fed with normal chow or 3-weeks DDC diet. Data presented as average ± SEM. * P < 0.05 compared to +DDC, *Rb*^*f/ f*^*; p53*^*f/f*^, # P < 0.05 compared to +DDC, *Rb*^Δ/Δ^, $ P < 0.05 compared to +DDC, *p53*^Δ/Δ^.

Serological evaluation of the alanine aminotransferase (ALT), an established marker for liver injury, revealed that ALT levels were strongly increased in mice fed with DDC (Fig 2A). Despite that *Rb*^Δ/Δ^;*p53*^Δ/Δ^ livers displayed less protoporphyrin accumulation and ductular reaction, their serum ALT levels were higher compared to other genotypes. To investigate whether increased hepatic injury was associated with enhanced cell death in *Rb*^Δ/Δ^;*p53*^Δ/Δ^ livers, we employed a terminal deoxynucleotidyl transferase–mediated deoxyuridine triphosphate nick end labeling (TUNEL) on liver sections (Fig 2B). Quantification of TUNEL positive liver cells revealed that all mice treated with DDC showed an increase in the number of dead hepatocytes compared to untreated mice. Hardly any TUNEL positive bile duct epithelial cells or oval cells were detected. Combined Rb and p53 loss in the liver resulted in a significant increase in hepatocellular death in mice fed DDC compared to other genotypes (Fig 2B and 2C). These findings indicate that Rb and p53 deficient livers experience less ductular reaction, but instead more hepatocellular injury under DDC exposure.

### Inactivation of Rb and p53 accelerates hepatocellular proliferation and polyploidization in response to DDC

Since *Rb*^Δ/Δ^;*p53*^Δ/Δ^ liver/body weight ratios were higher compared to *Wt* livers in mice that received a DDC diet for 3 weeks (S2B Fig), we investigated whether increased hepatocellular death in *Rb*^Δ/Δ^;*p53*^Δ/Δ^ livers was compensated by accelerating cell division and/or polyploidization of hepatocytes. To this end, we performed immunohistochemical analysis on liver sections with antibodies directed Ki67 (stained cells in cell cycle phase G1, S, G2/M) and phospho-histone 3 (PH3, stained cells in G2/M). In mice fed a normal chow, inactivation of Rb and p53 did not alter the proliferation rate of liver cells. In contrast, when mice were fed a DDC diet, *Rb*^Δ/Δ^;*p53*^Δ/Δ^ livers displayed a marked increase in mitotic figures, Ki67 and PH3 positive hepatocytes compared to *Wt*, *Rb*^Δ/Δ^ or *p53*^Δ/Δ^ livers (Fig 2B, 2D and 2E). Proliferation rates of bile duct cells and oval cells did not differ between the four different genotypes (S4A and S4B Fig). Furthermore, we observed that several *Rb*^Δ/Δ^;*p53*^Δ/Δ^ hepatocytes were multinucleated or contained extreme large nuclei, suggesting that these hepatocytes underwent enhanced polyploidization (Fig 2F). Two board certified veterinary pathologist have analyzed and scored these lesions. The anisokaryosis score, a measurement for differences in nuclear size, and the incidence of multinucleation were the highest in *Rb*^Δ/Δ^;*p53*^Δ/Δ^ livers (Fig 2F and 2G). Rb or p53 ablation alone already resulted in higher anisokaryosis and multinucleation, but to lesser degree than *Rb*^Δ/Δ^;*p53*^Δ/Δ^ livers. These findings suggest that the enhanced hepatocellular death in Rb and p53 deficient hepatocytes is compensated by increased proliferation and polyploidization of surrounding hepatocytes.

### Inactivation of Rb and p53 shortens life span and accelerates HCC formation after DDC exposure

**Fig 3:**
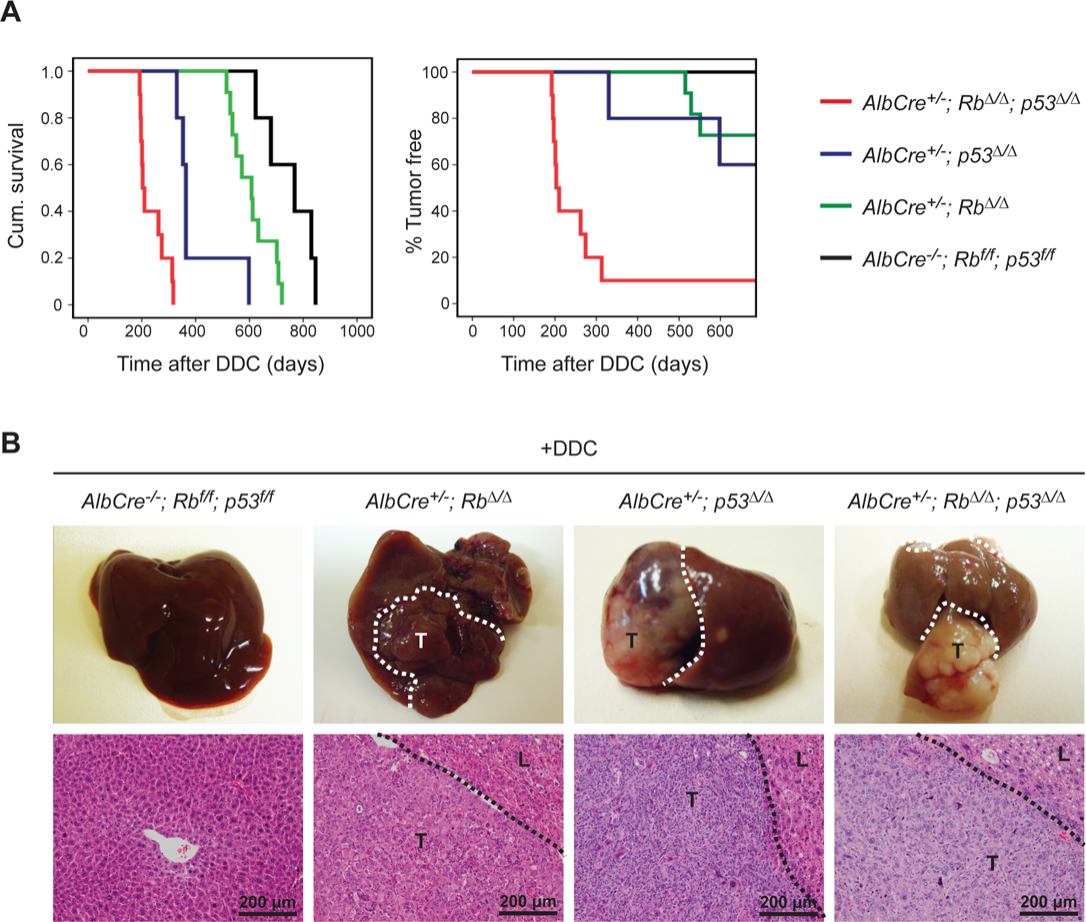
Inactivation of Rb and p53 shortens life span and accelerates hepatocellular carcinoma formation after DDC exposure. (**A**) Survival and time to tumor curves of mice of indicated genotypes after feeding with DDC diet for 3 weeks at young ages and aged with normal chow. (**B**) Representative macroscopic pictures of H&E staining of liver tumors of mice feeding with DDC diet for 3 weeks at young ages and aged with normal chow. White and black dotted lines indicate the borders between normal liver tissue (L) and tumor (T).

To determine whether increased hepatocellular death and proliferation in *Rb*^Δ/Δ^;*p53*^Δ/Δ^ livers after 3 weeks of DDC exposure had any long-term effects on liver homeostasis, mice were subsequently aged on a normal diet. Alb^+/-^; *Rb*^Δ/Δ^; *p53*^Δ/Δ^ mice needed to be sacrificed already after approximately 7.5 months after DDC feeding, because of severe swelling of the abdominal cavity. Alb^+/-^;*p53*^Δ/Δ^ mice lived longer, but needed to be sacrificed after approximately 15.5 months, whereas Alb^+/-^; *Rb*^Δ/Δ^ mice were euthanized at approximately 20 months after DDC exposure (Fig 3A; Table 1). Non-DDC treated Alb^+/-^; *Rb*^Δ/Δ^; *p53*^Δ/Δ^ mice had longer life span (mean survival time 527 days) (data not shown) compared to DDC treated Alb^+/-^; *Rb*^Δ/Δ^; *p53*^Δ/Δ^ mice (mean survival time 300 days). Necropsy of mice with swollen abdomen revealed the presence of visible liver neoplasm. Tumor incidences differ among groups, which were 27.3%, 40% and 90% in *Rb*^Δ/Δ^, *p53*^Δ/Δ^ and *Rb*^Δ/Δ^;*p53*^Δ/Δ^ livers, respectively (Fig 3A; Table 1). No liver tumors were observed in the age-matched control mice lacking *Alb-Cre*. Histologic examination of the liver samples was performed in a blinded fashion by a board certified veterinary pathologist. While livers harboring a single tumor suppressor loss were predominantly classified as well-differentiated HCC, livers deficient for both Rb and p53 were categorized as undifferentiated carcinoma (Fig 3B; Table 1). The results indicated that Rb and p53 have important tumor suppressor functions in hepatocytes after DDC-induced liver injury.

**Table 1:** Percentage tumor incidence, mean time to tumors and tumor classifications of mice with the indicated genotypes.

Genotype	% tumor incidence	Mean time to tumors (days)	% Well-differentiated HCC	% undifferentiated carcinoma
***AlbCre***^***-/-***^***; Rb***^***f/f***^***; p53***^***f/f***^	0% (0/5)	n.a.	n.a.	n.a.
***AlbCre***^***+/-***^***; Rb***^***Δ/Δ***^	27.3% (3/11)	600	100% (3/3)	0%
***AlbCre***^***+/-***^***; p53***^***Δ/Δ***^	40% (2/5)	464*	50% (1/2)	50% (1/2)
***AlbCre***^***+/-***^***; Rb***^***Δ/Δ***^***; p53***^***Δ/Δ***^	90% (9/10)	227*	22.2 (2/9)	77.8% (7/9)

*AlbCre*^*+/-*^*; p53*^*Δ/Δ*^ mice have a significant lower mean time to tumor compared to *AlbCre*^*+/-*^*; Rb*^*Δ/Δ*^ mice (*P<0.05); *AlbCre*^*+/-*^*; Rb*^*Δ/Δ*^*; p53*^*Δ/Δ*^ has a significant lower mean time to tumor compared to *AlbCre*^*+/-*^*; p53*^*Δ/Δ*^ (*P< 0.05; SPSS log rank test). n.a., not applicable.

## Discussion

Metabolism of xenobiotics occurs in mammals predominantly in the liver. Human patients with liver disease or liver cancer are treated frequently with xenobiotic drugs, and the efficacy of drug treatment depends on Cyp enzymes since they are required for xenobiotic metabolism. Our studies demonstrate that Rb and p53, two proteins often inactivated in liver diseases, are essential for Cyp expression. These findings are in line with studies demonstrating that loss of Rb and p53 results in reduced expression of a number of genes involved xenobiotic metabolism, such as Cyp3a11 and Cyp3a25 [3,4]. However, we show now that reduced expression of Cyp through liver-specific inactivation of Rb and p53 is accompanied by reduced metabolism of the xenobiotic DDC through evaluation of protoporphyrin, an *in vivo* measurable fluorescent product that accumulates in response to xenobiotic metabolism of DDC.

**Fig 4:**
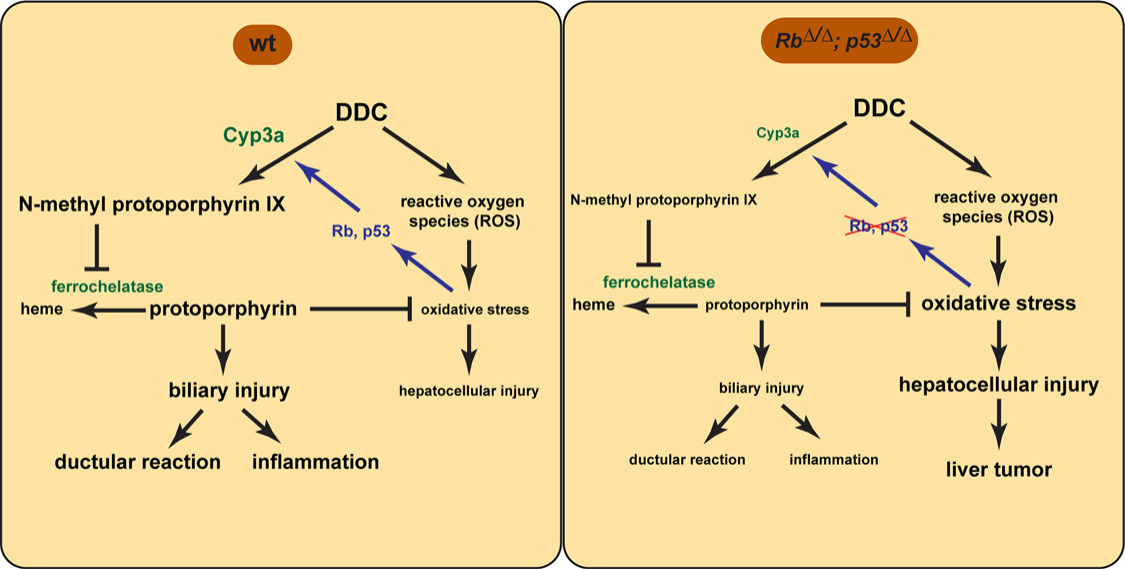
Scheme for the role of RB and p53 in DDC metabolism. DDC interacts with Cyp in particular Cyp3a to form *N*-methyl protoporphyrin IX which inhibits ferrochelatase causing an accumulation of protoporphyrin in the bile ducts. Bile duct injury induces ductular reaction and inflammation. In addition, DDC induces reactive oxygen species (ROS) and oxidative stress causing hepatocellular injury. Protoporphyrin, on the other hand, acts as an antioxidant and protects hepatocytes. Rb and p53 are important for Cyp expression. Oxidative stress activates Rb and p53. Loss of Rb and p53 results in reduced expression of Cyp3a, therefore less production of the ferrochelatase inhibitor and thereby prevents the accumulation of its substrate protophorphyrin. Consequently, less bile injury and ductular reaction was observed. The reduction of protoporphyrin, an antioxidant, increases oxidative induced hepatocellular injury and later liver tumors develop.

DDC interacts with Cyps in particular Cyp3as, proteins that contain a heme group [23]. During oxidative metabolism the 4-methyl group of DDC is transferred to one of pyrrole nitrogen of the heme group of Cyps leading to the formation of *N*-methyl protoporphyrin IX [22] (Fig 4). This *N*-methyl protoporphyrin IX then migrate from the site of production the endoplasmatic reticulum of hepatocytes to the inner membrane of the mitochondria to inhibit ferrochelatase, a key enzyme in the heme biosynthesis pathway. The mechanism by which *N*-methyl protoporphyrin IX inhibit ferrochelatase is thought to occur through competitive substrate inhibition with the endogenous protoporphyrin [22]. The block at this level of the heme biosynthesis causes marked accumulation of protoporphyrin in bile ducts [22,23] (Fig 4). Human patients with porphyria display a similar phenotype because they carry mutations in ferrochelatase gene [24,25]. Because DDC interacts directly with Cyp enzymes to generate the ferrochelatase inhibitor, reduced expression of Cyp enzymes in *Rb*^Δ/Δ^;*p53*^Δ/Δ^ livers would likely results in less production of the ferrochelatase inhibitor and thereby prevent the accumulation of its substrate protoporphyrin (Fig 4). Alternatively, loss of Rb and p53 could directly regulate the expression levels of ferrochetalase, but qPCR analysis revealed no changes in expression (Fig 1E), ruling out any direct regulation mechanism on ferrochelatase expression.

Increase protoporphyrin crystals induce injury to biliary epithelial cells. The injured biliary epithelial cells then secrete proinflammatory and chemotactic cytokines, such as TNF-α and IL-6, causing acute cholangitis, ductular reaction and finally biliary fibrosis [26]. Increase biliary pressure as a result of partial bile duct obstruction due to protoporphyrin plugs may be another mechanism that triggers ductular reaction in DDC model [15]. Since inactivation of Rb and p53 reduced the accumulation of protoporphyrin, consequently less biliary injury was observed and the degree of cholangitis and ductular reaction was dramatically decreased (Fig 4). Alternatively, Rb and p53 function might be required for the ductular reaction, especially for the proliferation of the liver progenitor cells. We have isolated and cultured liver progenitor cells utilizing the liver organoid technology [27], but we observed no differences in cell proliferation between Wt and Rb/p53 deficient liver progenitor cells (data not shown). These findings suggest that the reduced ductular reaction is not due to the fact that Rb and p53 are essential for liver progenitor cell proliferation, but rather due to reduced accumulation of protoporphyrin, the trigger for the biliary injury. Besides the reduced liver progenitor cells accumulation, we have observed reduced infiltration of inflammatory cells, which might be related to reduce biliary injury. However we cannot exclude that deletion of Rb and p53 in hepatocytes also resulted in a compromised immune response, because previous studies described that Rb potentiates the innate immune response in hepatocytes [28].

Although *Rb*^Δ/Δ^;*p53*^Δ/Δ^ livers displayed less biliary injury, hepatocellular injury was more severe, since ALT levels and the number of dead hepatocytes were increased. Loss of hepatocytes was partially compensated by increased cell division of surrounding hepatocytes as quantified by counting mitotic figures and immunohistochemical staining for the proliferation marker Ki67 and PH3. In addition, multiple Rb and p53 deficient hepatocytes were bigger in size and contained multiple nuclei or enlarged single nuclei, indicating that these cells are polyploid and that loss of hepatocytes was also partially compensated by increasing the size of surrounding hepatocytes.

Polyploidy, the addition of one multiple complete sets of chromosomes, starts in rodents after weaning and the degree of polyploidy increases with age [29]. However, pathological polyploidization can also be induced by DNA damage and oxidative stress [30,31]. Previous work demonstrated that deletion of Rb in the liver is associated with increased DNA damage and enhanced polyploidy in hepatocytes [32,33], which is consistent with our observation that liver specific deletion of Rb increases polyploidy. Inactivation of p53 also results in enhanced hepatocyte polyploidy, especially in response to partial hepatectomy [34]. Interestingly, in our study we found in the livers of DDC-fed mice that *p53* deletion resulted in the formation of hepatocytes with enlarged nuclei, and in addition we observed the formation of large multiple multinucleated cells with more than 2 nuclei. These findings indicate that p53 is important to prevent multinucleation in response hepatic injury. Combined deletion of Rb and p53 enhances this polyploid phenotype presumably through more DNA damage, because both tumor suppressors are important guardians of the genome. Moreover, metabolism of DDC by Cyp enzymes is associated with the generation of reactive oxygen species (ROS) and oxidative stress on hepatocytes [23], which is in line with our finding that DDC exposure enhances liver cell polyploidy. Noteworthy, protoporphyrin can act in a dark environment such as the liver as antioxidant by scavenging peroxyl radicals [23]. Since *Rb*^Δ/Δ^;*p53*^Δ/Δ^ livers show less protoporphyrin accumulation and consequently less antioxidants, the increase in hepatocellular injury and polyploidy might also be related to more oxidative stress (Fig 4).

Oxidative stress is known to mediate damage to cell structures including lipids, proteins and DNA [35]. The Rb and p53 tumor suppressor pathways are activated in response to DNA damage in order to block proliferation, induce apoptosis or enhance DNA repair [9–12]. Because DDC causes oxidative stress and presumably DNA damage, the tumor suppressor functions of Rb and p53 are most likely activated to protect the genome of hepatocytes. p53 activation most likely leads to activation of p21^Cip/Waf^ cyclin-dependent kinase inhibitor, resulting in a cell cycle arrest and possibly senescence [36]. Moreover, DNA damage has been shown to activate p16, another cyclin-dependent kinase inhibitor, which prevents Rb phosphorylation and inhibit E2F mediated transactivation [11]. However, inactivation of Rb or p53 alone had no major effect on hepatocellular proliferation or apoptosis in response to DDC, suggesting that Rb and p53 could partially compensate for each other functions. It has been shown that the p53 pathway could mediate compensatory cell cycle exit and apoptosis in the absence of Rb [37]. Lacking of both Rb and p53, DDC-induced injured hepatocytes continued to proliferate. Unrestricted hepatocyte proliferation under oxidative stress condition could result in genomic instability, which is supported by the increase in cell death and polyploidy after DDC exposure.

Long-term follow up studies revealed that these mice developed HCC even so DDC exposure was stopped after 3 weeks, indicating that loss of Rb and p53 in presence of DDC resulted in the formation of preneoplastic lesions that ultimately progressed to liver tumors. DDC exposure to wild-type mice did not result in spontaneous liver tumors. Individual deletion of Rb or p53 resulted in the formation of liver tumors as well, but with longer latency, lower frequency and less malignancy compared to combined deletion of Rb and p53. This less aggressive tumor phenotype of individual knockout mice corresponded with less cell death, less proliferation and less polyploidy of hepatocytes during DDC exposure. These studies are in line with studies showing that Rb and p53 suppress tumorigenesis in response to carcinogens [4,38,39]. However, here we demonstrate that a non-carcinogenic drug, such as DDC, can enhance tumorigenesis in livers deficient for Rb and p53.

In summary, we demonstrate that Rb and p53 are not only important for tumor suppression, but are also critical for xenobiotic metabolism in the liver. Based on the fact that Rb and p53 are frequently inactivated in liver cancers through hepatitis B or C virus infection, reduced xenobiotic metabolism in these livers could influence the efficacy or toxicity of therapeutic drugs or toxins respectively. Since Rb and p53 influence the expression of a large set of Cyp enzymes, evaluation of the status of Rb and p53 in the liver before treatment of human patients, might be important in order to adjust the therapeutic window of drugs.

## Supporting Information

S1 Fig Confirmation for genetic deletion of *Rb* and *p53* genes in liver tissue and tumors. Genotyping PCR for *Cre*, *Rb* and *p53* on normal livers (L) and liver tumors (T) from *Alb-cre* mice carrying conditional floxed alleles for *Rb*^*f/f*^ and *p53* ^*f/f*^ alleles. ^Δ^ stands for deleted allele.
(TIF)

S2 Fig Mice fed with DDC have yellowish mucous membrane, darker livers and increase liver weight. (**A**) *In situ* view of mucous membrane and livers in the abdomen, and images of cut livers of mice after 3 weeks of DDC diet or control (normal chow). (**B**) Percentage of liver weight to body weight of mice after 3 weeks of DDC diet or control (normal chow).
(TIF)

S3 Fig Macrophages and hepatic stellate cells were reduced upon loss of Rb and p53. (**A**) Quantitative analysis of macrophages number per high power field (HPF) and representative pictures of F4/80 staining in mice of indicated genotypes after 3 weeks of DDC. (**B**) Quantitative analysis of hepatic stellate cells number per high power field (HPF) and representative pictures of GFAP staining in mice of indicated genotypes after 3 weeks of DDC.
(TIF)

S4 Fig Proliferation rates of bile duct cells and oval cells did not differ between the four different genotypes. (**A**) Percentage of PH3 positive nuclei of bile duct cells and (**B**) oval cells from mice fed with normal chow or DDC diet for 3 weeks. Data presented as average ± SEM.
(TIF)

S1 File Materials and Methods. (DOCX)

S1 Table Primer sequences used for RT-PCR. (DOCX)
