# Supplementary figures and images for: Rb and p53 Liver Functions Are Essential for Xenobiotic Metabolism and Tumor Suppression
Source: PLoS One. 2016 Mar 11;11(3):e0150064. doi: 10.1371/journal.pone.0150064 (PMC4788452; doi:10.1371/journal.pone.0150064)

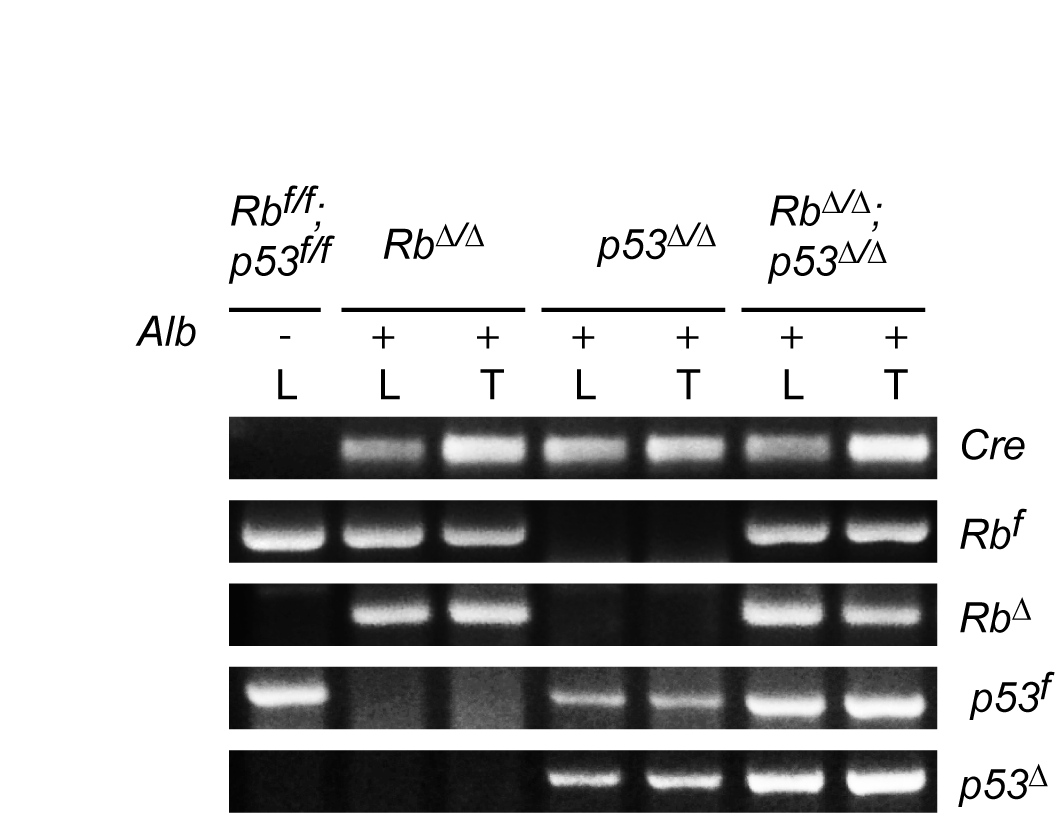

Supplement: S1 Fig — Genotyping PCR for Cre, Rb and p53 on normal livers (L) and liver tumors (T) from Alb-cre mice carrying conditional floxed alleles for Rbf/f and p53 f/f alleles. Δ stands for deleted allele. (TIF) [file pone.0150064.s001.tif]

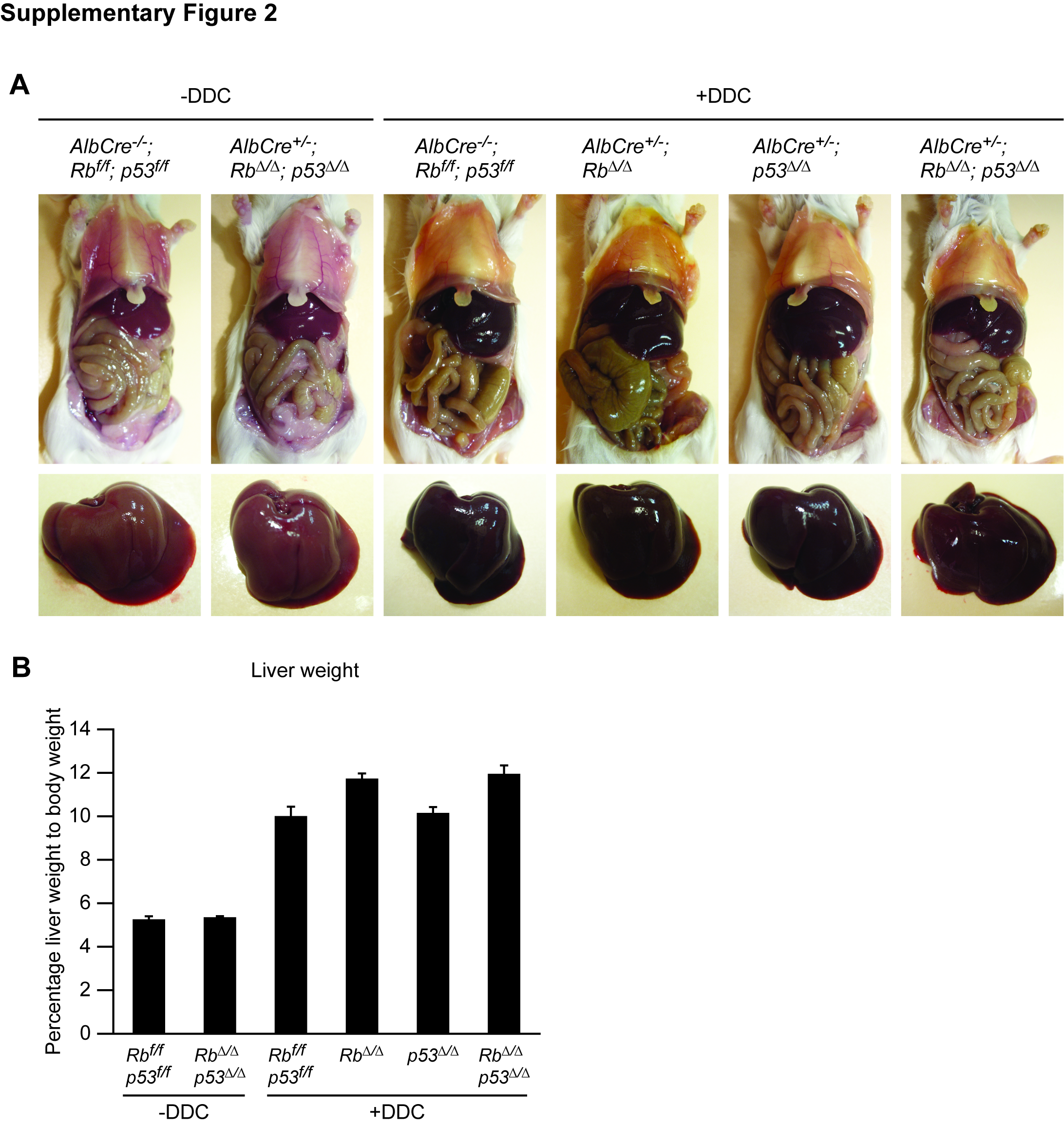

Supplement: S2 Fig — (A) In situ view of mucous membrane and livers in the abdomen, and images of cut livers of mice after 3 weeks of DDC diet or control (normal chow). (B) Percentage of liver weight to body weight of mice after 3 weeks of DDC diet or control (normal chow). (TIF) [file pone.0150064.s002.tif]

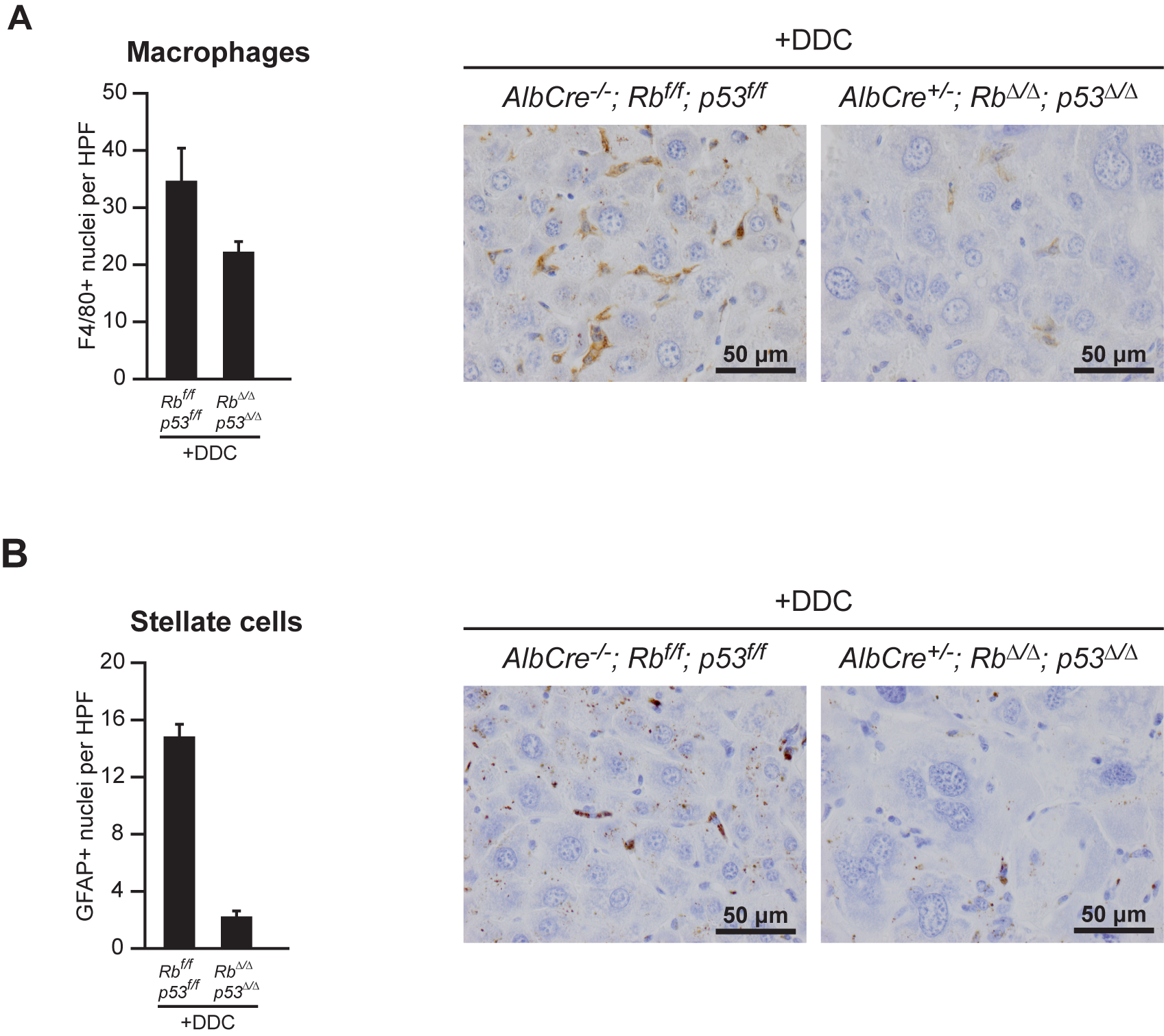

Supplement: S3 Fig — (A) Quantitative analysis of macrophages number per high power field (HPF) and representative pictures of F4/80 staining in mice of indicated genotypes after 3 weeks of DDC. (B) Quantitative analysis of hepatic stellate cells number per high power field (HPF) and representative pictures of GFAP staining in mice of indicated genotypes after 3 weeks of DDC. (TIF) [file pone.0150064.s003.tif]

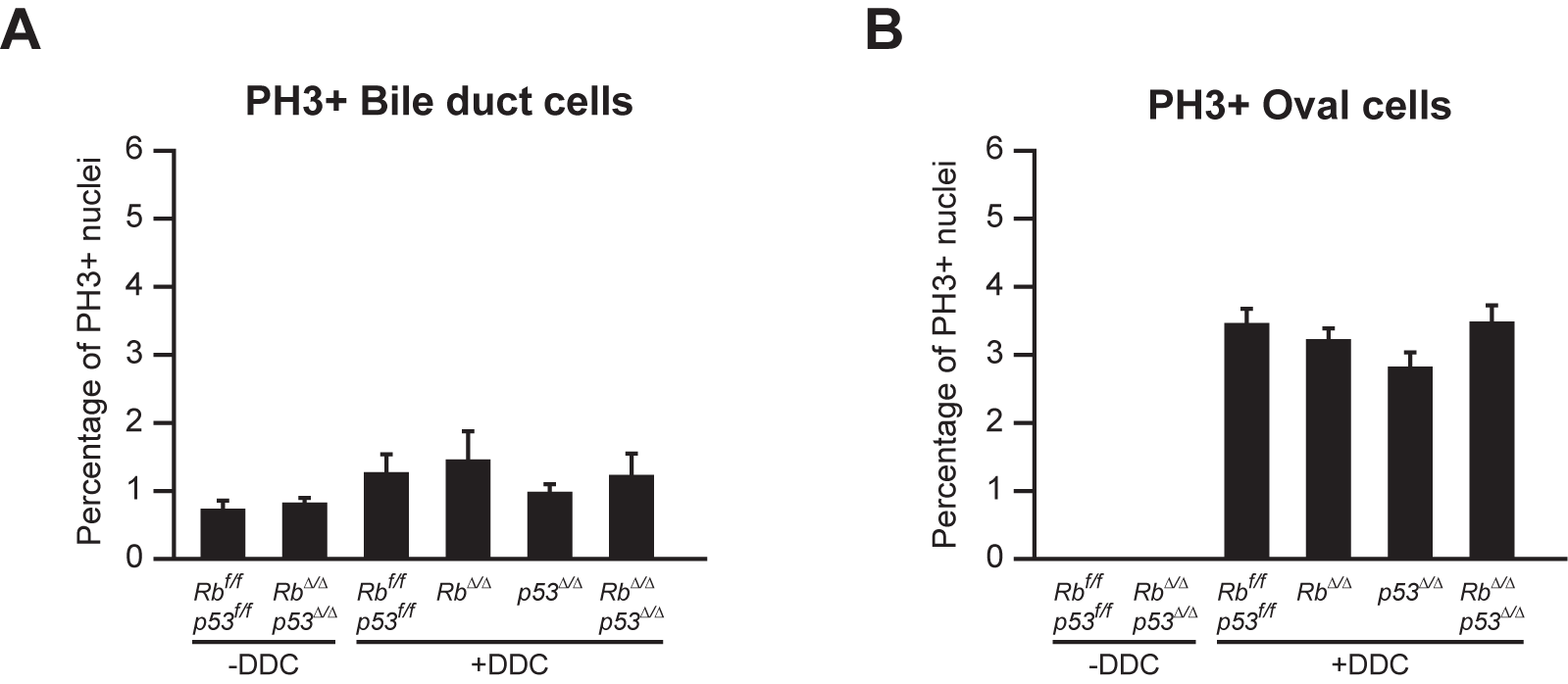

Supplement: S4 Fig — (A) Percentage of PH3 positive nuclei of bile duct cells and (B) oval cells from mice fed with normal chow or DDC diet for 3 weeks. Data presented as average ± SEM. (TIF) [file pone.0150064.s004.tif]
