# Supplementary material for: Rb and p53 Liver Functions Are Essential for Xenobiotic Metabolism and Tumor Suppression
Source: PLoS One. 2016 Mar 11;11(3):e0150064. doi: 10.1371/journal.pone.0150064 (PMC4788452; doi:10.1371/journal.pone.0150064)
Supplement: S1 File — (DOCX) [file pone.0150064.s005.docx]

**Supporting materials and methods**

**Immunohistochemistry**

A portion of excised liver was fixed in 10% formalin overnight and then processed for paraffin embedding. 4-µm thick section was deparaffinized in xylene and rehydrated in 100%, 96%, 70% alcohol and distilled water respectively. Antigen retrieval was done by heating the slides in 10mmol/liter citrate buffer pH 6.0 for 15 minutes at 70% microwave power, followed by cooling down for 30 minutes at room temperature. The endogenous peroxidase activity was blocked by 1% H2O2 solution (except 3% H2O2 solution for CK19) for 30 minutes. After 15 minutes treating with normal goat serum, the slides were incubated at 4°C overnight with antibodies against Ki67 (Biogenex, Fremont), PH3 (Upstate, Temecula), CK19 (Kindly gift from AMC, Amsterdam), F4/80 (Abcam, Cambridge) and GFAP (NeoMarkers, Cheshire). The sections were incubated with suitable concentration of biotin-labelled secondary antibodies (Vector labs, Burlingame) for 30 minutes, and subsequently with Vectastain Elite ABC reagents (Vector labs, Burlingame) for 30 minutes. The reaction was visualized by incubating with DAB solution for 10 minutes. TUNEL staining was done according to the manufacturer’s instruction (Millipore, Darmstadt). The slides were then counterstained with hematoxylin for 30 seconds, and covered with coverslips utilizing Eukitt (Sigma-Aldrich, Zwijndrecht).

Percentage of TUNEL and PH3 hepatocytes were quantified by counting positive hepatocytes from total of 1000 hepatocytes. Mitotic index and multinucleated hepatocytes were counted for 10 high power field (HPF) and 5 x20 fields, respectively. Anisokaryosis score was performed from 10 x20 fields.

**Pathological analysis of liver tissues**

Pathological analysis was performed by two board-certified veterinary pathologists. Liver tumors were classified according to the nomenclature and diagnostic criteria for hepatobiliary lesions in rats and mice (1).

**Quantitative PCR Analysis**

RNA was isolated from frozen liver tissues using Qiagen RNeasy kit according to the manufacturer’s instruction. Then, complementary DNA was generated by reverse transcriptase with random hexamer primers (RevertAid™ First Strand cDNA Synthesis Kit, Fermentas, Waltham). Duplo quantitative PCR was performed on BioRad MyiQ Cycler and visualized using SYBRgreen supermix (BioRad, Veenendaal). Quantification of gene expression was determined by the delta delta Ct method and normalized to *18S* and *beta-actin* housekeeping genes. Specific primer details for each gene are shown in supplementary table 1.

**Genotype analysis**

Genotyping on livers and liver tumors was performed using allele-specific primers. The following primers have been used: *Rb*F: CTCAAGAGCTCAGACTCATGG, *Rb*R: GGCGTGTGCCATCAATG, *Rb*212: GAAAGGAAAGTCAGGGACATTGGG, *p53*F: AAGGGGTATGAGGGACAAGG, *p53*R: GAAGACAGAAAAGGGGAGGG, *p53*F1(int): CACAAAAACAGGTTAAACCCA, *Cre*F: ATGCTTCTGTCCGTTTGCCG, *Cre*R: CCTGTTTTGCACGTTCACCG;

**Reference**

(1) Thoolen B, Maronpot RR, Harada T, Nyska A, Rousseaux C, Nolte T, et al. Proliferative and nonproliferative lesions of the rat and mouse hepatobiliary system. Toxicol Pathol 2010 Dec;38(7 Suppl):5S-81S.
