# Supplementary material for: Rb and p53 Liver Functions Are Essential for Xenobiotic Metabolism and Tumor Suppression
Source: PLoS One. 2016 Mar 11;11(3):e0150064. doi: 10.1371/journal.pone.0150064 (PMC4788452; doi:10.1371/journal.pone.0150064)
Supplement: S1 Table — (DOCX) [file pone.0150064.s006.docx]

**Supporting table**

**S1 Table Primer sequences used for RT-PCR**

| **Gene** | **Direction** | **Sequence** |
| --- | --- | --- |
| *CK19* | Forward | 5’-CGGTGGAAGTTTTAGTGGGACC-3’ |
|  | Reverse | 5’-CTTCTCATTGCCAGACAGCAGC-3’ |
| *Cyp3a11* | Forward | 5’-TCTCCTTGCTGTCACAGACCC-3’ |
|  | Reverse | 5’-CATCTCCTTGAGTTTTCCACTGG-3’ |
| *FECH* | Forward | 5’-TGTTTTCTGCCCACTCCCTG-3’ |
|  | Reverse | 5’-ACTGCCAAACCAGTCGGTAGG-3’ |

*CK19*: Cytokeratin 19; *Cyp3a11*: Cytochrome P450 3a11; *FECH*: ferrochelatase
